# Translating neuronal activity at the synapse: presynaptic calcium sensors in short-term plasticity

**DOI:** 10.3389/fncel.2014.00356

**Published:** 2014-10-29

**Authors:** Arthur P. H. de Jong, Diasynou Fioravante

**Affiliations:** ^1^Department of Neurobiology, Harvard Medical SchoolBoston, MA, USA; ^2^Department of Neurobiology, Physiology and Behavior, Center for Neuroscience, University of California DavisDavis, CA, USA

**Keywords:** C2 domain, protein kinase C, Munc13, synaptotagmin, calmodulin, post-tetanic potentiation, residual calcium, short-term plasticity

## Abstract

The complex manner in which patterns of presynaptic neural activity are translated into short-term plasticity (STP) suggests the existence of multiple presynaptic calcium (Ca^2+^) sensors, which regulate the amplitude and time-course of STP and are the focus of this review. We describe two canonical Ca^2+^-binding protein domains (C2 domains and EF-hands) and define criteria that need to be met for a protein to qualify as a Ca^2+^ sensor mediating STP. With these criteria in mind, we discuss various forms of STP and identify established and putative Ca^2+^ sensors. We find that despite the multitude of proposed sensors, only three are well established in STP: Munc13, protein kinase C (PKC) and synaptotagmin-7. For putative sensors, we pinpoint open questions and potential pitfalls. Finally, we discuss how the molecular properties and modes of action of Ca^2+^ sensors can explain their differential involvement in STP and shape net synaptic output.

## Introduction

Synaptic transmission is initiated by action potential-evoked influx of calcium (Ca^2+^) into the presynaptic terminal, which triggers fusion of vesicles by binding to a specialized Ca^2+^ sensor. Bursts of action potentials lead to the buildup of residual Ca^2+^ ([Ca^2+^]_residual_) in the terminal, which outlives neuronal activity, and induce multiple forms of short-term presynaptic plasticity (STP), including facilitation, depression, augmentation and post-tetanic potentiation (PTP) (reviewed in Fioravante and Regehr, 2011). STP plays a crucial role in synaptic computations and shapes the properties of microcircuits (reviewed in Abbott and Regehr, 2004; Regehr, 2012).

**Figure 1:**
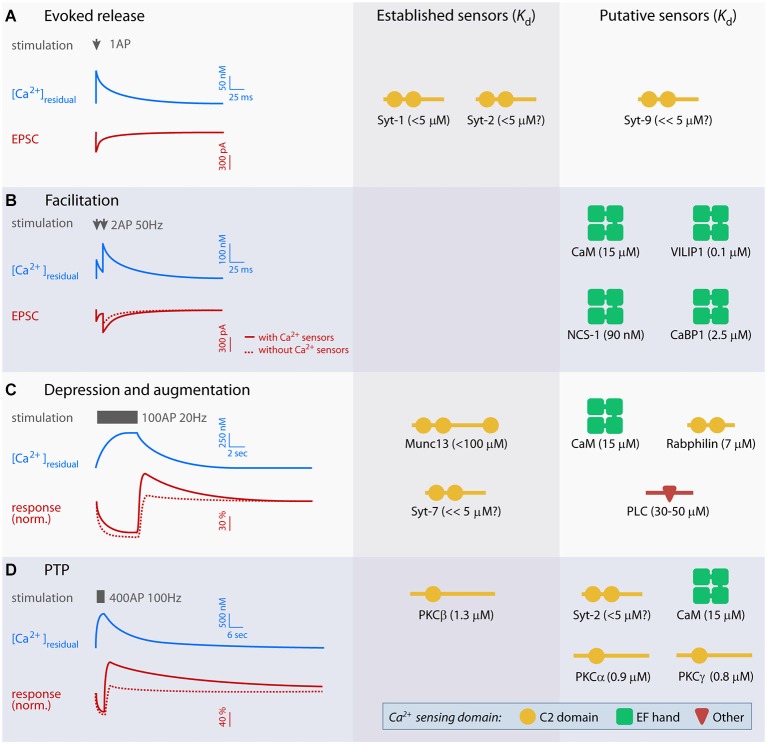
**Overview of established and putative presynaptic Ca^2+^ sensors in evoked release and short-term plasticity (STP)**. Left panel displays idealized traces of [Ca^2+^]_residual_ and excitatory postsynaptic currents (EPSCs; **A** and **B**) or baseline-normalized responses (**C** and **D**) during synaptic plasticity based on experiments at parallel fiber synapses, mossy fiber-CA3 synapses, the crayfish neuromuscular junction and the calyx of Held. Typical stimulation paradigms used to elicit various forms of STP are indicated in gray. Scale bars are approximate, but note that the amplitude and kinetics of the Ca^2+^ signal and STP vary significantly between preparations. Right panels show established and putative Ca^2+^ sensors for evoked release **(A)** and each form of STP **(B–D)**, and their Ca^2+^ dissociation constant (K_d_). K_d_ values were obtained from: syt-1 C2AB (with PIP2) (van den Bogaart et al., 2012), free calmodulin (CaM; Xia and Storm, 2005), visin-like protein (VILIP-1) (myristroylated) (Li et al., 2011), neuronal calcium sensor 1 (NCS-1) (myristroylated) and CaBP1 (Aravind et al., 2008), Munc13 C2B (Shin et al., 2010), Rabphilin C2B (Ubach et al., 1999), PLCδ1 (Grobler and Hurley, 1998) PKCα, -β and γ (Torrecillas et al., 2004). The K_d_ values of syt-2, -7 and -9 have not been measured directly, but indirect measurements suggest that syt-2 is similar to syt-1, whereas syt-7 and 9 may have lower K_d_ (Sugita et al., 2002).

The dynamics of some forms of STP are dictated by the kinetics of [Ca^2+^]_residual_ (Delaney et al., 1989; Kamiya and Zucker, 1994) and can be explained by changes in vesicular release probability (Katz and Miledi, 1968; Zucker and Stockbridge, 1983) or by depletion of the readily releasable pool (RRP) of vesicles (Bailey and Chen, 1988; Liu and Tsien, 1995; von Gersdorff and Matthews, 1997). However, at several synapses the magnitude of facilitation is higher than can be explained by [Ca^2+^]_residual_ alone, and both facilitation and PTP decay slower than the [Ca^2+^]_residual_ signal (Regehr et al., 1994; Atluri and Regehr, 1996; Brager et al., 2003; Felmy et al., 2003; Fioravante et al., 2011; Figure 1). Furthermore, many types of STP rely on the regulation of steps upstream of vesicle fusion (Dittman and Regehr, 1998; Wang and Kaczmarek, 1998), including RRP refilling and Ca^2+^ influx through voltage-gated Ca^2+^ channels (VGCCs; Stevens and Wesseling, 1998; Xu and Wu, 2005; Mochida et al., 2008; Müller et al., 2008; Leal et al., 2012). These events are strongly Ca^2+^-dependent, and thus Ca^2+^ sensors must be activated to induce and sustain STP. The Ca^2+^ sensors that mediate STP are the topic of this mini-review. First, we will discuss the molecular structure and function of two Ca^2+^-binding domains employed by Ca^2+^ sensors: C2 domains and EF-hands. Subsequently, we will define the criteria for establishing Ca^2+^ sensors for STP, and, guided by these criteria, discuss a body of recent literature on well accepted and putative sensors that regulate STP.

## Ca^2+^-binding motifs

### C2 domains

The best described Ca^2+^ sensors in the context of synapses are C2 domains, which are found in many signal transduction and membrane trafficking proteins (Rizo and Südhof, 1998). C2 domains consist of ~130 amino acids that form a compact β-sandwich of two 4-stranded β-sheets. Three loops connecting the β-sheets at the top of the domain contain 4–5 highly conserved aspartates that coordinate the binding of 2 to 3 Ca^2+^ ions (Shao et al., 1996; Ubach et al., 1998; Fernandez et al., 2001). The Ca^2+^-binding properties of C2 domains have been described in detail in synaptotagmin (syt), which acts as the Ca^2+^ sensor for synchronous vesicle fusion at most synapses (Pang and Südhof, 2010). Mutations that interfere with Ca^2+^ binding on syt-1 alter the Ca^2+^-sensitivity of vesicle fusion (Nishiki and Augustine, 2004; Shin et al., 2009; Kochubey and Schneggenburger, 2011; Kochubey et al., 2011). Analogous mutation analyses of other C2 domains showed similar effects on Ca^2+^ binding (Shin et al., 2010; Fioravante et al., 2014; Liu et al., 2014). Some C2 domains naturally lack these aspartate residues and cannot bind Ca^2+^ (e.g., Pappa et al., 1998). Ca^2+^ binding increases the affinity of C2 domains for phospholipids (Brose et al., 1992; Fernandez et al., 2001), thus recruiting the domain to the plasma membrane. In addition, it may trigger a conformational change that increases association with effector proteins (for instance syt-1 binding to SNAREs (Bai et al., 2004)) or exposes a domain within the protein (e.g., the MUN domain of Munc13 (Shin et al., 2010)). Many C2 domains display a steep increase in Ca^2+^ affinity in the presence of phosphatidylinositol 4,5-biphosphate (PIP2; van den Bogaart et al., 2012), which helps localize the domain to the PIP2-enriched active zone (Rohrbough and Broadie, 2005).

### EF-hands

The EF-hand is the most common Ca^2+^-binding motif, with diverse cellular functions including cytoplasmic Ca^2+^ buffering and signal transduction (Skelton et al., 1994; Schaub and Heizmann, 2008; Schwaller, 2009). The motif consists of two α-helices connected by a linker of 12 amino acids (Lewit-Bentley and Réty, 2000). Six residues within this linker coordinate binding to a single Ca^2+^ ion, and their mutation abolishes Ca^2+^ binding (Maune et al., 1992). Examples of EF-hand-containing proteins with proposed Ca^2+^-sensing roles in STP include calmodulin (CaM), neuronal calcium sensor 1 (NCS-1) and visin-like proteins (VILIPs).

CaM is the prototypical EF-hand protein that interacts with numerous effector proteins in a Ca^2+^-dependent manner (Xia and Storm, 2005). Important presynaptic effectors are CaM-dependent kinase II (CaMKII), myosin light chain kinase (MLCK), adenylyl cyclase, the protein phosphatase calcineurin, Munc13, VGCCs and Ca^2+^-activated potassium channels, all of which regulate presynaptic function (de Jong and Verhage, 2009; Adelman et al., 2012). Because the Ca^2+^ affinity of CaM is differentially regulated by its binding partners, different CaM-protein complexes vary in their Ca^2+^ sensitivity (Olwin and Storm, 1985; Xia and Storm, 2005) and could therefore be differentially engaged during various forms of STP. Direct assessment of the role of CaM as a Ca^2+^ sensor for STP has proven difficult because manipulations of CaM levels alter expression of >200 genes (Pang et al., 2010) and rescue experiments in neuronal preparations with Ca^2+^-binding mutants of CaM have not been conducted thus far.

## Definition of a Ca^2+^ sensor for STP

With a plethora of C2- and EF-hand-containing proteins in the presynaptic terminal, there are numerous candidate Ca^2+^ sensors for STP. We propose that in order to qualify as a sensor for STP, a protein must fulfill the following three criteria:

1. *Ca^2+^ must bind directly to the protein*. An obvious requirement for a Ca^2+^ sensor is that it must bind Ca^2+^. Some EF-hands and C2 domains lack the Ca^2+^-coordinating residues and cannot bind Ca^2+^. Therefore, Ca^2+^ binding must be experimentally established for each protein.
2. *Protein must be part of, or directly modulate, vesicle availability or the vesicle release machinery*. Changes in availability and/or fusogenicity of synaptic vesicles and in presynaptic Ca^2+^ influx shape STP (Dutta Roy et al., 2014). A Ca^2+^ sensor for STP must therefore directly affect vesicle availability (recruitment, docking, priming) and/or the vesicle fusion machinery, including VGCCs and SM proteins (for a discussion of release machinery, see Südhof, 2013). This definition includes enzymes like kinases, which directly regulate the properties of these components. For the purpose of this review, we do not consider Ca^2+^ buffers (e.g., parvalbumin) and pumps, which indirectly affect STP by changing the spatiotemporal distribution of free Ca^2+^ through binding or extrusion (Müller et al., 2007; Scullin and Partridge, 2010), or components of the endocytotic machinery, which can affect vesicle or release site availability after prolonged episodes of exocytosis (Wilkinson and Lin, 2004; Hosoi et al., 2009).
3. *Mutations that interfere with Ca^2+^binding affect STP*. Even if a protein satisfies criteria 1 and 2, it is not a Ca^2+^ sensor for STP unless Ca^2+^ binding is required for the protein’s function in STP. For instance, whether Ca^2+^ binding to Doc2 is required for spontaneous release is debated and the role of Doc2 as a Ca^2+^ sensor for spontaneous release remains unclear (Groffen et al., 2010; Pang et al., 2011). Therefore, it is necessary to show that mutation of the Ca^2+^ binding site abolishes function (for example using a knockout/rescue or knockin approach) in order to conclude that a protein is a Ca^2+^ sensor mediating STP. It could even be argued that a requirement for Ca^2+^ binding *during plasticity* must be demonstrated in order to establish a protein as a Ca^2+^ sensor, but the technology for this type of experiments is currently lacking.

## Ca^2+^ sensors in STP

### Facilitation

At synapses with low initial release probability, brief bursts of activity can induce transient facilitation of release, which relies on increased release probability due to elevated [Ca^2+^]_residual_ (Katz and Miledi, 1968; Kamiya and Zucker, 1994; Regehr et al., 1994). However, this mechanism alone cannot fully explain the magnitude of facilitation at all synapses (Atluri and Regehr, 1996; Felmy et al., 2003), and additional Ca^2+^-dependent processes have been suggested (Zucker and Regehr, 2002), including the existence of a yet unidentified presynaptic Ca^2+^ sensor distinct from syt-1 (Bain and Quastel, 1992; Saraswati et al., 2007). Enhancement of Ca^2+^ currents is an attractive mechanism to mediate facilitation, and the capability of Ca^2+^/CaM to modulate overexpressed VGCCs during strong depolarization has been studied extensively (Catterall et al., 2013). Ca^2+^/CaM binds to a regulatory domain of Ca_v_2.1, the VGCC that mediates the P/Q type Ca^2+^ current driving synaptic transmission in most synapses. In heterologous cell lines, this interaction leads to enhancement of Ca^2+^ currents, which depends on Ca^2+^ binding to CaM (Lee et al., 1999; DeMaria et al., 2001). Several EF-hand-containing proteins including VILIPs, CaBPs and NCS-1 (collectively named neuronal Ca^2+^ sensors, or nCaS) also modulate Ca^2+^ influx through VGCCs (Few et al., 2005; Lautermilch et al., 2005; Burgoyne, 2007; Dason et al., 2012; Catterall et al., 2013) and may affect facilitation in a manner that depends on the nCaS binding domain of VGCCs (Tsujimoto et al., 2002; Sippy et al., 2003; Mochida et al., 2008; Leal et al., 2012). For none of these protein functions, however, has a Ca^2+^ binding requirement been established, and some of them may actually be independent of Ca^2+^ (Few et al., 2005). In addition, due to the lack of suitable genetic models, most experiments rely on overexpression of exogenous proteins (Mochida et al., 2003). Whether nCaS are specifically involved in the regulation of STP, or the altered STP is a consequence of altered basal synaptic properties, remains controversial (Dason et al., 2012).

### Depression and recovery from depression

Prolonged high-frequency stimulation leads to transient decrease in presynaptic strength, which can be due to depletion of the RRP (Elmqvist and Quastel, 1965; Liu and Tsien, 1995; Schneggenburger et al., 2002) and activity-dependent decrease in Ca^2+^ influx (Forsythe et al., 1998; Xu and Wu, 2005) (for a complete review of known mechanisms of depression, see Regehr, 2012). CaM, CaBP1 and NCS-1 have been proposed as putative Ca^2+^ sensors to mediate the latter effect (Xu and Wu, 2005; Catterall and Few, 2008; Mochida et al., 2008). Depression can be slowed by Ca^2+^-dependent replenishment of the RRP (Stevens and Wesseling, 1998; Wang and Kaczmarek, 1998). The vesicle priming factor Munc13 acts as a Ca^2+^ sensor to determine the rate of depression, via its C2B and CaM-binding domains. Ca^2+^ binding to the C2B domain of Munc13 activates its MUN domain that promotes assembly of the machinery responsible for vesicle fusion, thereby increasing refilling of the RRP (Shin et al., 2010; Ma et al., 2011). Indeed, Munc13 knockout neurons expressing a variant of the protein with mutated Ca^2+^-coordinating aspartates display increased synaptic depression without affecting initial release probability (Shin et al., 2010). In addition, Munc13 binds Ca^2+^/CaM, and this interaction also accelerates RRP refilling (Junge et al., 2004; Lipstein et al., 2012, 2013). In line with this observation, CaM inhibitors slow the RRP refilling rate (Sakaba and Neher, 2001; Hosoi et al., 2007). Although a Ca^2+^-binding CaM mutant has not been studied in this context, the CaM/Munc13 interaction is strongly Ca^2+^-dependent (Junge et al., 2004; Dimova et al., 2006; Lipstein et al., 2012), thus making the Ca^2+^/CaM-Munc13 complex a likely Ca^2+^-sensor for STP.

Synaptotagmin-7 has also been identified as a sensor that regulates depression, operating via its two Ca^2+^-binding C2 domains (Liu et al., 2014). At the zebrafish neuromuscular junction, syt-7 regulates desynchronized release (Wen et al., 2010), but its function in mammalian neurons has been debated (Maximov et al., 2008; Bacaj et al., 2013; Liu et al., 2014). A recent study showed that in syt-7 knockout mice, initial release probability is unaffected but the rate of vesicle replenishment during and after bursts of activity is significantly reduced (Liu et al., 2014). This phenotype is rescued by wild-type syt-7 but not by syt-7 carrying mutations of the Ca^2+^ binding sites, demonstrating that syt-7 is a Ca^2+^ sensor that mediates RRP refilling. Syt-7 also probably interacts with Ca^2+^/CaM (Liu et al., 2014), but the functional significance of this complex remains to be identified.

In contrast to the proteins discussed above that promote recovery from depression, rabphilin is thought to slow down recovery from depression (Deák et al., 2006). Rabphilin is a synaptic vesicle protein with two Ca^2+^-sensing C2 domains (Yamaguchi et al., 1993; Ubach et al., 1999; Coudevylle et al., 2008), but whether Ca^2+^ binding is required for its role in STP has not been determined.

### Augmentation and PTP

Augmentation and PTP are two closely related forms of STP that require prolonged high-frequency stimulation (Magleby, 1973; Magleby and Zengel, 1976a; Stevens and Wesseling, 1999; Habets and Borst, 2005; Korogod et al., 2005). For augmentation, varying stimulus duration increases the peak amplitude of the enhancement without significantly affecting the time course of decay (Magleby, 1979). The mechanisms underlying augmentation are not well understood and changes in both release probability and Ca^2+^-dependent replenishment of the RRP have been proposed (Magleby and Zengel, 1976b; Stevens and Wesseling, 1999; Rosenmund et al., 2002; Kalkstein and Magleby, 2004). Munc13 and syt-7 have been suggested as Ca^2+^ sensors for augmentation (Shin et al., 2010; Lipstein et al., 2013; Liu et al., 2014), but since both sensors affect depression as well, dissociation of their roles in synaptic depression vs. augmentation has not been possible. Various phospholipase C (PLC) isoforms could also act as Ca^2+^ sensors because they require binding of a Ca^2+^ ion for activation of their catalytic domain (Grobler and Hurley, 1998; Rebecchi and Pentyala, 2000). Pharmacological studies suggest that PLC activation is required for augmentation (Rosenmund et al., 2002) but not PTP (Genc et al., 2014). PLC hydrolyses PIP2 to diacylglycerol, which could lead to potentiation of synaptic transmission via Munc13 and protein kinase C (PKC; de Jong and Verhage, 2009).

PTP typically lasts longer than augmentation and shows a progressive increase in the time course of decay with increased duration and frequency of stimulation (Magleby, 1979; Korogod et al., 2005). Pharmacological (e.g., Alle et al., 2001; Brager et al., 2002; Beierlein et al., 2007; Korogod et al., 2007) and genetic (Fioravante et al., 2011, 2012, 2014; Chu et al., 2014) studies at several synapses have firmly established the requirement for PKC in PTP. Three PKC isoforms (α, β and γ) possess a C2 domain and bind Ca^2+^ with low micromolar affinity (Torrecillas et al., 2004; Newton, 2010; Figure 1). PKCs enhance release through phosphorylation of effectors, including components of the vesicular release machinery such as Munc18 (Wierda et al., 2007; de Jong and Verhage, 2009; Genc et al., 2014). Mutations of the Ca^2+^-coordinating aspartates in the C2 domain of PKCβ abolish its ability to support PTP, without affecting basal synaptic function (Fioravante et al., 2014).

PKCβ is probably not the only Ca^2+^ sensor for PTP. At the immature calyx of Held, PTP depends on PKCγ (Chu et al., 2014). Moreover, at the parallel fiber-Purkinje cell synapse in the cerebellum, PKCα can readily support PTP in the absence of PKCβ and γ (Fioravante et al., 2012). It remains to be tested whether Ca^2+^ binding to PKCα and γ is necessary for PTP and whether all PKC isoforms act through Munc18 phosphorylation. Finally, pharmacological studies suggest that Ca^2+^/CaM, acting via MLCK, makes a small contribution to PTP at immature, but not functionally mature, synapses (Lee et al., 2008; Fioravante et al., 2011).

Tetanic stimulation enhances not only evoked responses (i.e., PTP) but also spontaneous events in a Ca^2+^-dependent manner. The frequency (Zengel and Magleby, 1981; Zucker and Lara-Estrella, 1983; Eliot et al., 1994; Habets and Borst, 2005), and at some synapses also the amplitude (He et al., 2009), of spontaneous events increase after tetanization. Because of similarities in the time course of these effects with PTP, a common mechanism has been speculated (Zengel and Magleby, 1981). However, the effects of [Ca^2+^]_residual_ on spontaneous transmission were recently shown to be independent of PKC (Xue and Wu, 2010; Fioravante et al., 2011; but see Brager et al., 2003) and the increase in amplitude requires syt-2 (He et al., 2009). The Ca^2+^ sensors remain unknown.

## Differential engagement of Ca^2+^ sensors and implications for STP

Different patterns of neuronal activity result in variable Ca^2+^ signals stretching over an order of magnitude (Figure 1). Diverse sensors are therefore needed to translate the Ca^2+^ signals into distinct forms of STP. Factors such as Ca^2+^ affinity, specific (sub-)cellular expression and mechanisms of action contribute to the specialization of sensors for different forms of STP. For example, NCS-1 has high affinity for Ca^2+^ and localizes at the plasma membrane (O’Callaghan et al., 2002; Burgoyne, 2007) where it could rapidly respond to local Ca^2+^ signals. PKCβ, on the other hand, has lower Ca^2+^ affinity, is cytoplasmic at rest (Newton, 2010) and likely has to phosphorylate more than one substrates to induce plasticity; therefore, sustained, global Ca^2+^increases are likely required for its activation, in agreement with the prolonged stimulation requirement for PTP (Habets and Borst, 2005; Korogod et al., 2005). Even for the same sensor, Ca^2+^ affinity can vary as a result of effector binding, phospholipid binding, and post-translational modifications (Xia and Storm, 2005; Li et al., 2011; van den Bogaart et al., 2012). Finally, specific expression patterns of Ca^2+^ sensors could help explain why identical activation regimes do not always lead to the same STP across synapses or during development (Rosenmund et al., 2002; Chu et al., 2014).

Most synapses exhibit multiple forms of STP and the net synaptic output reflects the interaction between these different forms (de Jong and Verhage, 2009). It is therefore likely that different Ca^2+^ sensors interact, and might even compete (Chu et al., 2014), during STP. The dynamics of these interactions should be considered when building computational models of STP. Traditionally, such models combine use-dependent depletion and Ca^2+^-dependent facilitation to explain synaptic output (Tsodyks et al., 1998; Fuhrmann et al., 2002; Pfister et al., 2010). Introduction of additional components such as vesicle replenishment, which are engaged under conditions that activate the corresponding Ca^2+^ sensors, more accurately reflects our understanding of the underlying biology and allows better prediction of synaptic and network behavior (Hennig, 2013).

## Conflict of interest statement

The authors declare that the research was conducted in the absence of any commercial or financial relationships that could be construed as a potential conflict of interest.
